# Critical genomic insights into vancomycin-resistant *Enterococcus faecium* in Lebanon

**DOI:** 10.1128/spectrum.00171-25

**Published:** 2025-08-05

**Authors:** Marwan Osman, Iman Yassine, Jouman Hassan, Jessica E. Ericson, Steven J. Schiff, Anahita Ghorbani Tajani, Bledar Bisha, Monzer Hamze, Issmat I. Kassem

**Affiliations:** 1 Department of Neurosurgery, Yale University School of Medicine 5755, New Haven, Connecticut, USA; 2 Laboratoire Microbiologie, Santé et Environnement (LMSE), Doctoral School of Sciences and Technology, Faculty of Public Health, Lebanese University, Tripoli, Lebanon; 3 Nuffield Department of Population Health, University of Oxford 6396, Oxford, United Kingdom; 4 Department of Food Science and Technology, Center for Food Safety, University of Georgia 92569, Griffin, Georgia, USA; 5 Department of Pediatrics, Penn State College of Medicine 12310 , Hershey, Pennsylvania, USA; 6 Department of Epidemiology of Microbial Diseases, Yale University School of Public Health 50296 , New Haven, Connecticut, USA; 7 Department of Animal Science, University of Wyoming 4416, Laramie, Wyoming, USA; 8 Clinical Laboratory, Microbiology Department, Nini Hospital 237177, Tripoli, Lebanon; 9 Faculty of Agricultural and Food Sciences, American University of Beirut 11238, Beirut, Lebanon; Universidad Nacional Autonoma de Mexico - Campus Morelos, Cuernavaca, Mexico

**Keywords:** vancomycin-resistant *Enterococcus faecium*, CC17, genomic epidemiology, antimicrobial resistance, surveillance, One Health, public health, whole genome sequencing

## Abstract

Vancomycin-resistant *Enterococcus faecium* (VRE) poses a global public health threat, with particularly severe ramifications in low-resource settings. While *E. faecium* is commonly a commensal organism in the human gut, the acquisition of vancomycin resistance has contributed to the emergence of VRE as a major cause of hospital-acquired infections, especially in vulnerable populations. This study investigates the genetic characteristics of VRE isolated from hospitalized patients in Lebanon, focusing on antimicrobial resistance (AMR) phenotypes and the underlying resistome. We collected VRE isolates from patients at the time of admission or during routine screenings for multidrug-resistant pathogens. The isolates were identified using matrix-assisted laser desorption/ionization time-of-flight mass spectrometry (MALDI-TOF MS) and screened for antimicrobial susceptibility using the disk diffusion and E-test methods. Whole genome sequencing was conducted using Illumina technology, and bioinformatics analyses were carried out to identify the resistome, sequence types, plasmid types, and virulence genes. Additionally, we analyzed previously GenBank-deposited VRE genomes from Lebanon to assess genetic relationships. Our VRE isolates carried multiple acquired AMR genes and chromosomal mutations, exhibiting resistance to vancomycin, telavancin, ampicillin, erythromycin, norfloxacin, levofloxacin, and nitrofurantoin. The isolates belonged to three subtypes: ST203 (67%), ST612 (22%), and a novel ST2711 (11%), all of which belonged to CC17, a globally prevalent hospital-associated lineage known for high levels of AMR and hospital outbreaks. Core-genome single nucleotide variants (SNVs) phylogenetic analysis revealed that the ST203 isolates clustered with the previously GenBank-deposited ST203 Lebanese genomes. Taken together, these findings highlight the growing VRE burden in low-resource countries, emphasizing the need for enhanced surveillance and alternative treatment strategies.

**IMPORTANCE:**

The healthcare system in Lebanon faces substantial challenges due to ongoing economic instability and limited resources, creating a conducive environment for the spread of antimicrobial resistance. Vancomycin-resistant *Enterococcus faecium* (VRE) represents a growing public health threat, yet studies addressing its genetics in Lebanon are scarce. This study provided critical insights into the genomic features of VRE in clinical settings, highlighting the potential clonal dissemination of multidrug-resistant isolates carrying the *vanHAX* operon and belonging to CC17, a globally prevalent, hospital-associated lineage. Resistance to novel antimicrobials, such as telavancin, dalbavancin, and eravacycline, is alarming and necessitates immediate action to preserve these critical last-resort interventions. Notably, the identification of clonal spread and a novel sequence type (ST2711) within Lebanese hospitals, alongside resistance to last-resort antimicrobials, highlights the dynamic evolution of VRE and the urgent need to strengthen antimicrobial stewardship programs in Lebanon and beyond. Addressing this challenge requires integrated One Health strategies.

## OBSERVATION

Disadvantaged communities in low-income countries are particularly vulnerable to multidrug-resistant (MDR) infections due to limited access to quality healthcare services, suboptimal living conditions, and insufficient infection control measures, among other factors (1, 2). These significant challenges are further compounded by the growing problem of antimicrobial resistance (AMR), which threatens to undermine the effectiveness of treatment options and increases the burden of infectious diseases (3–5).

Vancomycin-resistant *Enterococcus faecium* (VRE) is classified as a serious AMR threat by the Centers for Disease Control and Prevention, highlighting its significance in healthcare and community settings (6). VRE infections are challenging to treat and are associated with high mortality rates, severe morbidity, and considerable healthcare costs, especially in resource-limited settings (7). Although nine acquired *van* gene clusters have been identified in *E. faecium*, vancomycin resistance is primarily driven by the acquisition of *vanA* and *vanB* (8). These two genes are the most prevalent, presumably due to their frequent association with mobile genetic elements. Notably, VanA-type VRE displays high-level resistance to vancomycin and teicoplanin, while VanB-type VRE is resistant only to vancomycin (9). Furthermore, a main vancomycin resistance mechanism is mediated by the *vanHAX* operon, which encodes enzymes that modify the peptidoglycan precursor terminus from D-Ala-D-Ala to D-Ala-D-Lac. This structural modification reduces vancomycin binding affinity by approximately 1,000-fold, leading to clinically significant resistance to glycopeptides (10).

Despite its growing clinical significance, VRE remains a neglected issue in Lebanon, with limited research and awareness about its prevalence and impact. Only two retrospective nationwide studies, based on annual institutional antimicrobial susceptibility testing (AST) reports, have described an increase in VRE prevalence from 0% in 2011 to 2% in 2016 (11). Additionally, 53 *E. faecium* genomes from Lebanon are available in PubMLST; however, these genomes have not been analyzed in terms of antimicrobial resistance and virulence. Furthermore, their relationship to other strains has neither been discussed nor evaluated in the scientific literature. To date, comprehensive studies on VRE, including genomic analyses of VRE isolates, are lacking in the country. This knowledge gap complicates efforts to understand VRE’s burden and manage this resistant pathogen effectively. To address this issue, this study aimed to delineate the genetic background and underlying resistance mechanisms of VRE isolates from hospitalized patients who were either screened upon admission or during routine assessments for MDR pathogens at the Nini Hospital. This major facility serves around one million people in North Lebanon.

Between November 2019 and June 2022, samples from patients were spread onto Bile-Esculin agar (Bio-Rad, Hercules, California) plates, supplemented with 6 µg/mL vancomycin (Sigma-Aldrich, St. Louis, Missouri). After incubation for 24 h at 35°C under aerobic conditions, potential VRE colonies were selected and purified. The bacterial identification was further confirmed using matrix-assisted laser desorption/ionization time-of-flight mass spectrometry (MALDI-TOF MS) (Vitek MS, bioMérieux, Marcy L’Etoile, France). Nine VRE clinical isolates were identified and included in this study. Of these, seven isolates were obtained from stool samples, one from urine, and one from an axillary site. Notably, stool-associated VRE isolates have been recovered after a routine fecal screening for MDR pathogens, which is consistently performed on patients at admission at Nini Hospital as part of the internal antimicrobial stewardship guidelines. The isolates were recovered from different patients aged 63 to 98 years old. Eight isolates were identified during admission screening, suggesting a potential community origin, while one isolate was associated with healthcare exposure (Table S1).

Antimicrobial susceptibility patterns of the isolates were assessed using the Kirby Bauer disc diffusion and E-test assays as described in the guidelines of the Clinical and Laboratory Standards Institute (CLSI-M100). All the isolates exhibited resistance to vancomycin, telavancin, ampicillin, erythromycin, norfloxacin, levofloxacin, and nitrofurantoin, which belonged to more than three antimicrobial classes, confirming that the isolates were MDR. Moreover, most isolates were resistant to teicoplanin (7/9 isolates), dalbavancin (8/9), gentamicin (8/9), tetracycline (8/9), doxycycline (6/9), tigecycline (6/9), and eravacycline (6/9). Notably, intermediate resistance was observed against fosfomycin, chloramphenicol, and quinupristin-dalfopristin. None of the isolates exhibited resistance to daptomycin or linezolid (Table S2). Whole genome sequencing (WGS) was performed using the Illumina NGS platforms, and various bioinformatics tools were applied to investigate the isolates' resistome, virulome, pathogenicity, and multi-locus sequence types (MLST) (Supplemental text). The genomic analysis of VRE isolates using Resfinder v.4.5 revealed the presence of 7–9 acquired resistance genes and numerous chromosomal mutations, consistent with the phenotypic AMR findings (Table 1). Taken together, our findings raise significant concerns regarding the management of VRE in clinical settings and beyond. The occurrence of these resistant strains among vulnerable patient populations within hospital environments is worrisome because treatment options are becoming severely limited. Indeed, resistance to novel antimicrobials, such as telavancin, dalbavancin, and eravacycline, is of particular concern and necessitates immediate action to preserve these critical last-resort treatments. Since VRE can show resistance/tolerance to disinfection procedures (12), early detection of VRE-colonized or infected patients is crucial to prevent transmission to other patients and the hospital environment (13).

**TABLE 1:** Clinical and genomic characterization of *E. faecium* isolates collected in this study

Sample ID	Accession number	AMR profile based on the disk diffusion assay^*a*^	AMR profile based on the E-test assay^*a*^	Sequence type (Clonal complex)^*b*^	Acquired antimicrobial resistance genes^*c*^	Quinolone resistance-determining region (QRDR) and PBP-5 mutations^*c*^	Virulence genes^*d*^	Human pathogenicity (%)^*e*^	Plasmid replicons detected by WGS (identity %)^*f*^
EF142	SRR26810637	**R:** AMP-VAN-ERY-TET-DOX-TGC^*g*^-ERV^*g*^-NOR-LVX-NIT **IR:** CHL **S:** FOS^*h*^-LZD	**R:** TLV^*i*^-DAL-TEC-GMN **IR:** QDA **SDD:** DAP^*j*^	203 (CC17)	*aac(6')-Ii; ant (6)-Ia; aac(6')-aph(2''); aph(2'')-Ia; msr(C); erm(B); cat(pC221); tet(M); vanHAX*	*gyrA*(S83Y); *parC*(S80R) *pbp5*(V24A); *pbp5*(S27G); *pbp5*(R34Q); *pbp5*(G66E); *pbp5*(A68T); *pbp5*(E85D); *pbp5*(E100Q); *pbp5*(K144Q); *pbp5*(T172A); *pbp5*(L177I); *pbp5*(D204G); *pbp5*(A216S); *pbp5*(T324A); *pbp5*(M485A); *pbp5*(N496K); *pbp5*(A499T); *pbp5*(E525D); *pbp5*(E629V); *pbp5*(P667S)	*acm; IS*16*; bepA; fms13; fms14; fnm; gls20; gls33; glsB; glsB1; orf1481; ptsD; sagA; sgrA*	88.2	rep11a (100%); rep7a (100%); repUS15 (99.81%); rep1 (100%); rep2 (100%)
EF321	SRR26810636	**R:** AMP-VAN-ERY-TET-DOX-TGC-ERV-NOR-LVX-NIT- **IR:** CHL **S:** FOS-LZD	**R:** TLV-DAL-TEC-GMN **SDD:** DAP **S:** QDA	203 (CC17)	*aac(6')-Ii; aac(6')-aph(2''); aph(2'')-Ia; ant (6)-Ia; msr(C); erm(B); cat(pC221); tet(M); vanHAX*	*gyrA*(S83Y); *parC*(S80R) *pbp5*(V24A); *pbp5*(S27G); *pbp5*(R34Q); *pbp5*(G66E); *pbp5*(A68T); *pbp5*(E85D); *pbp5*(E100Q); *pbp5*(K144Q); *pbp5*(T172A); *pbp5*(L177I); *pbp5*(D204G); *pbp5*(A216S); *pbp5*(T324A); *pbp5*(M485A); *pbp5*(N496K); *pbp5*(A499T); *pbp5*(E525D); *pbp5*(E629V); *pbp5*(P667S)	*acm; IS*16*; bepA; empA; empC; fms11; fms13; fms14; fms16; fms19; fnm; gls20; gls33; glsB; glsB1; orf1481; ptsD; sagA; sgrA*	88.2	rep7a (100%); rep1 (100%); rep2 (100%); repUS15 (99.81%); rep11a (100%)
EF901	SRR26810635	**R:** AMP-VAN-FOS-ERY-TET-DOX-TGC-ERV-NOR-LVX-NIT **S:** CHL-LZD	**R:** TLV-GMN **SDD:** DAP **S:** DAL-TEC-QDA	612 (CC17)	*aac(6')-Ii; ant (6)-Ia; aac(6')-aph(2''); msr(C); erm(T); tet(M); tet(L); dfrG; vanHAX*	*gyrA*(S83Y)*; parC*(S80I) *pbp5*(V24A); *pbp5*(S27G); *pbp5*(R34Q); *pbp5*(G66E); *pbp5*(A68T); *pbp5*(E85D); *pbp5*(E100Q); *pbp5*(K144Q); *pbp5*(T172A); *pbp5*(L177I); *pbp5*(D204G); *pbp5*(A216S); *pbp5*(T324A); *pbp5*(M485A); *pbp5*(N496K); *pbp5*(A499T); *pbp5*(E525D); *pbp5*(E629V); *pbp5*(P667S)	*acm; hyl_Efm_; IS*16*; empA; empB; fms13; fnm; gls20; gls33; glsB; glsB1; orf1481; ptsD; sagA; scm; sgrA*	79.6	rep18a (98.89%); repUS15 (99.71%); repUS43 (100%); repUS12 (99.62%)
EF1180	SRR26810643	**R:** AMP-VAN-ERY-TET-DOX-TGC-ERV-NOR-LVX-NIT **IR:** FOS **S:** CHL-LZD	**R:** TLV-DAL-TEC-GMN **SDD:** DAP **S:** QDA	203 (CC17)	*aac(6')-Ii; aac(6')-aph(2''); aph(2'')-Ia; aph(3')-III; ant (6)-Ia; msr(C); erm(B); tet(M); vanHAX*	*gyrA*(S83Y); *parC*(S80R) *pbp5*(V24A); *pbp5*(S27G); *pbp5*(R34Q); *pbp5*(G66E); *pbp5*(A68T); *pbp5*(E85D); *pbp5*(E100Q); *pbp5*(K144Q); *pbp5*(T172A); *pbp5*(L177I); *pbp5*(D204G); *pbp5*(A216S); *pbp5*(T324A); *pbp5*(M485A); *pbp5*(N496K); *pbp5*(A499T); *pbp5*(E525D); *pbp5*(E629V); *pbp5*(P667S)	*acm; IS*16*; bepA; empA; empC; fms11; fms13; fms14; fms16; fms19; fnm; gls20; gls33; glsB; glsB1; orf1481; ptsD; sagA; sgrA*	89.6	rep17 (100%); repUS15 (99.81%); rep2 (100%); rep11a (100%)
EF1196	SRR26810642	**R:** AMP-VAN-ERY-TET-DOX-NOR-LVX-NIT **IR:** FOS **S:** TGC-ERV-CHL-LZD-	**R:** TLV-DAL-TEC-GMN **IR:** QDA **SDD:** DAP	203 (CC17)	*aac(6')-Ii; aac(6')-aph(2''); aph(2'')-Ia; aph(3')-III; ant (6)-Ia; msr(C); erm(B); tet(M); vanHAX*	*gyrA*(S83Y); *parC*(S80R) *pbp5*(V24A); *pbp5*(S27G); *pbp5*(R34Q); *pbp5*(G66E); *pbp5*(A68T); *pbp5*(E85D); *pbp5*(E100Q); *pbp5*(K144Q); *pbp5*(T172A); *pbp5*(L177I); *pbp5*(D204G); *pbp5*(A216S); *pbp5*(T324A); *pbp5*(M485A); *pbp5*(N496K); *pbp5*(A499T); *pbp5*(E525D); *pbp5*(E629V); *pbp5*(P667S)	*acm; IS*16*; bepA; empA; empC; fms11; fms13; fms14; fms16; fms19; fnm; gls20; gls33; glsB; glsB1; orf1481; ptsD; sagA; sgrA*	89.6	rep11a (100%); rep2 (100%); rep17 (100%); repUS15 (99.81%)
EF1261	SRR26810641	**R:** AMP-VAN-ERY-NOR-LVX-NIT **IR:** CHL **S:** FOS-TET-DOX-TGC-ERV-LZD	**R:** TLV-DAL-TEC-GMN **SDD:** DAP **S:** QDA	2711 (CC17)	*aac(6')-Ii; ant (6)-Ia; aac(6')-aph(2''); aph(2'')-Ia; msr(C); erm(B); cat(pC221); vanHAX*	*gyrA*(S83Y); *parC*(S80R) *pbp5*(V24A); *pbp5*(S27G); *pbp5*(R34Q); *pbp5*(G66E); *pbp5*(A68T); *pbp5*(E85D); *pbp5*(E100Q); *pbp5*(K144Q); *pbp5*(T172A); *pbp5*(L177I); *pbp5*(D204G); *pbp5*(A216S); *pbp5*(T324A); *pbp5*(M485A); *pbp5*(N496K); *pbp5*(A499T); *pbp5*(E525D); *pbp5*(E629V); *pbp5*(P667S)	*acm; IS*16*; empA; empC; fms13; fms14; fnm; gls20; gls33; glsB; glsB1; orf1481; ptsD; sagA; sgrA*	88.2	rep1(100%); rep2 (100%); repUS7 (99.75%); repUS15 (98.81%); rep14b (100%); rep7a (100%); rep11a (100%)
EF1276	SRR26810640	**R:** AMP-VAN-ERY-TET-DOX-NOR-LVX-NIT **IR:** FOS **S:** TGC-ERV-CHL-LZD	**R:** TLV-DAL-TEC-GMN **SDD:** DAP **S:** QDA	203 (CC17)	*aac(6')-Ii; aac(6')-aph(2''); aph(2'')-Ia; aph(3')-III; ant (6)-Ia; msr(C); erm(B); tet(M); vanHAX*	*gyrA*(S83Y); *parC*(S80R) *pbp5*(V24A); *pbp5*(S27G); *pbp5*(R34Q); *pbp5*(G66E); *pbp5*(A68T); *pbp5*(E85D); *pbp5*(E100Q); *pbp5*(K144Q); *pbp5*(T172A); *pbp5*(L177I); *pbp5*(D204G); *pbp5*(A216S); *pbp5*(T324A); *pbp5*(M485A); *pbp5*(N496K); *pbp5*(A499T); *pbp5*(E525D); *pbp5*(E629V); *pbp5*(P667S)	*acm; IS*16*; bepA; empA; empC; fms11; fms13; fms14; fms16; fms19; fnm; gls20; gls33; glsB; glsB1; orf1481; ptsD; sagA; sgrA*	89.6	rep11a (100%); rep17 (100%); repUS15 (99.81%); rep2 (100%)
EF1334	SRR26810639	**R:** AMP-VAN-ERY-TET-DOX-TGC-ERV-NOR-LVX-NIT **IR:** FOS **S:** CHL-LZD	**R:** TLV-DAL-GMN **IR:** TEC **SDD:** DAP **S:** QDA	612 (CC17)	*aac(6')-Ii; ant (6)-Ia; aac(6')-aph(2''); msr(C); erm(T); tet(M); tet(L); dfrG; vanHAX*	*gyrA*(S83Y)*; parC*(S80I) *pbp5*(V24A); *pbp5*(S27G); *pbp5*(R34Q); *pbp5*(G66E); *pbp5*(A68T); *pbp5*(E85D); *pbp5*(E100Q); *pbp5*(K144Q); *pbp5*(T172A); *pbp5*(L177I); *pbp5*(D204G); *pbp5*(A216S); *pbp5*(T324A); *pbp5*(M485A); *pbp5*(N496K); *pbp5*(A499T); *pbp5*(E525D); *pbp5*(E629V); *pbp5*(P667S)	*acm; hyl_Efm_; IS*16*; empA; empB; fms13; fnm; gls20; gls33; glsB; glsB1; orf1481; ptsD; sagA; scm; sgrA*	82.5	repUS12 (99.62%); repUS15 (99.71%); rep18a (99.89%); repUS43 (100%)
EF1337	SRR26810638	**R:** AMP-VAN-ERY-TET-DOX-TGC-ERV-NOR-LVX-NIT **IR:** CHL **S:** FOS-LZD	**R:** TLV-DAL-TEC **SDD:** DAP **S:** QDA-GMN	203 (CC17)	*aac(6')-Ii; ant (6)-Ia; msr(C); erm(B); cat(pC221); tet(M); vanHAX*	*gyrA*(S83Y); *parC*(S80R) *pbp5*(V24A); *pbp5*(S27G); *pbp5*(R34Q); *pbp5*(G66E); *pbp5*(A68T); *pbp5*(E85D); *pbp5*(E100Q); *pbp5*(K144Q); *pbp5*(T172A); *pbp5*(L177I); *pbp5*(D204G); *pbp5*(A216S); *pbp5*(T324A); *pbp5*(M485A); *pbp5*(N496K); *pbp5*(A499T); *pbp5*(E525D); *pbp5*(E629V); *pbp5*(P667S)	*acm; IS*16*; bepA; empA; empC; fms11; fms13; fms14; fms16; fms19; fnm; gls20; gls33; glsB; glsB1; orf1481; ptsD; sagA; sgrA*	88.2	rep11a (100%); rep1 (100%); rep2 (100%); rep7a (100%); repUS15 (99.81%)

^*a*^ Resistance to antimicrobials was determined using the disk diffusion and E-test assays according to the Clinical and Laboratory Standards Institute (CLSI-M100) guidelines (https://clsi.org/standards/products/microbiology/companion/using-m100/). R, resistance; IR, intermediate resistance; SDD, susceptible-dose dependent; S, susceptible; AMP, ampicillin; VAN, vancomycin; FOS, Fosfomycin; ERY, erythromycin; TET, tetracycline; DOX, doxycycline; TGC, tigecycline; ERV, eravacycline; CHL, chloramphenicol; LZD, Linezolid; NOR, norfloxacin, LVX, Levofloxacin; NIT, Nitrofurantoin; GMN, Gentamicin; TLV, Telavancin; DAL, Dalbavancin; TEC, Teicoplanin; DAP, Daptomycin; QDA, Quinupristin-dalfopristin.

^*b*^ Sequence type (ST) was determined by MLSTFinder v2.0.9 (https://cge.food.dtu.dk/services/MLST/).

^*c*^ Acquired antimicrobial drug resistance genes detected by ResFinder v4.5.0 (http://genepi.food.dtu.dk/resfinder) using the default thresholds of 90% minimum identity and 60% minimum coverage.

^*d*^ Virulence genes detected by VirulenceFinder v2.0 (https://cge.food.dtu.dk/services/VirulenceFinder/) using 100% minimum identity and 100% minimum coverage.

^*e*^ Using PathogenFinder v1.1 (https://cge.food.dtu.dk/services/PathogenFinder/), *Enterococcus faecium isolates* were predicted to be a human pathogen.

^*f*^ Plasmid types were detected by PlasmidFinder v2.1 (https://cge.food.dtu.dk/services/PlasmidFinder/) using the default thresholds of 95% minimum identity and 60% minimum coverage.

^*g*^ The CLSI guidelines do not provide clinical breakpoints for tigecycline and eravacycline; therefore, the clinical breakpoints were adopted from the European Committee on Antimicrobial Susceptibility Testing (EUCAST) guidelines.

^*h*^ According to the CLSI guidelines, fosfomycin should only be reported on *Enterococcus faecalis*.

^*i*^ According to the CLSI guidelines, there is no intermediate or resistant category for telavancin, dalbavancin; any non-susceptible results are classified as resistant.

^*j*^ The CLSI guidelines on daptomycin breakpoints indicate that there is no longer a "susceptible" category for *E. faecium*. Instead, only SDD and R breakpoints are available.

The isolates belonged to three subtypes: ST203 (66.7% of the isolates), ST612 (22.2%), and a novel ST2711 (11.1%). Notably, resistance gene profiles varied between these STs. The *aph(2″)-Ia*, *erm(B*), and *cat(pC221*) genes were observed in most ST203 and ST2711 isolates but not in any ST612 isolates, while the *erm(T*), *tet(L*), and *dfrG* genes were exclusively found in ST612 isolates. Additionally, in ST203 and ST2711 isolates, the *vanHAX* operon is present on small contigs (~5–11 kb), with most isolates harboring IS*1251* preceding the operon. This arrangement suggests an association with Tn*1546-like* transposons, although the small contig sizes prevent determining whether these elements are plasmid- or chromosome-borne. The ST612 isolates carried the vancomycin resistance operon on larger contigs (~26 kb) with a complete Tn*1546* structure, but the absence of plasmid-specific genes leaves their genomic location unresolved. This limitation reflects the inherent constraints of short-read sequencing, which preclude the resolution of the replicon origin. Further experiments using long-read sequencing and hybrid assembly approaches will be necessary to resolve plasmid structures and facilitate a comprehensive analysis of their association with antimicrobial susceptibility and resistome profiles.

**Fig 1:**
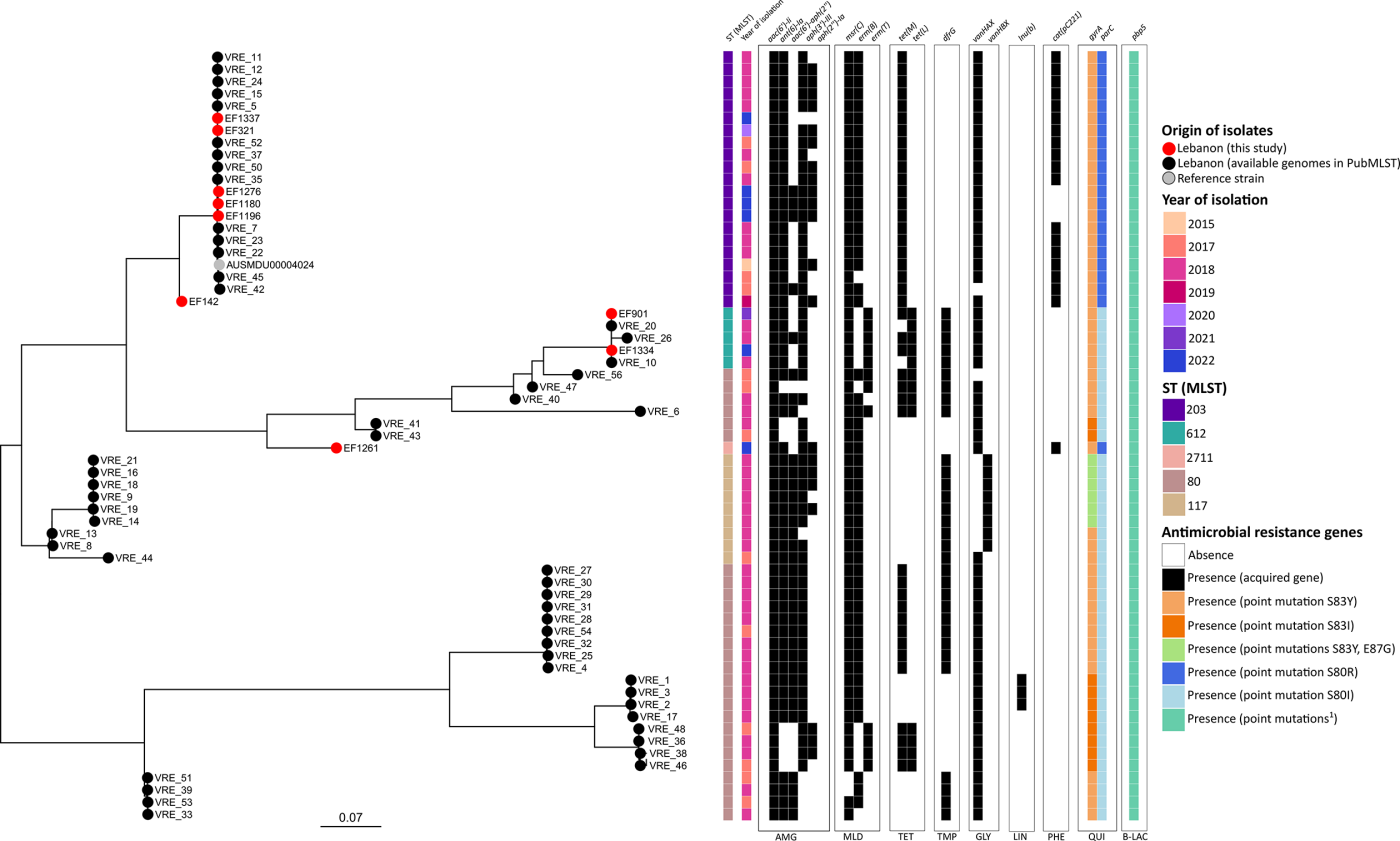
Core-genome phylogenetic tree of our collected *Enterococcus faecium* isolates (*n* = 9) and all Lebanese *E. faecium* genomes available in GenBank Lebanon (*n* = 53). All isolates belong to clonal complex 17 (CC17). ST2711 is a novel subtype. The strips from left to right show the year of isolation, sequence type (ST), antimicrobial resistance genes, and the point mutations in *gyrA*, *parC*, and *pbp5* genes detected in their whole-genome sequences. ^1^*pbp5* mutations are *pbp5*(V24A), *pbp5*(S27G), *pbp5*(R34Q), *pbp5*(G66E), *pbp5*(A68T), *pbp5*(E85D), *pbp5*(E100Q), *pbp5*(K144Q), *pbp5*(T172A), *pbp5*(L177I), *pbp5*(D204G), *pbp5*(A216S), *pbp5*(T324A), *pbp5*(M485A), *pbp5*(N496K), *pbp5*(A499T), *pbp5*(E525D), *pbp5*(E629V), and *pbp5*(P667S). AMG, aminoglycosides; MLD, macrolides; TET, tetracyclines; TMP, folate antagonist; GLY, glycopeptides; LIN, lincosamides; PHE, amphenicols; QUI, quinolones; B-LAC, β-lactams.

All STs belong to clonal complex 17 (CC17), a globally prevalent, hospital-associated lineage recognized for its high levels of AMR and significant role in hospital outbreaks (14–16). To further explore the genetic relationships, we also analyzed previously GenBank-deposited genomes from Lebanon, all of which also belonged to CC17. Core-genome single nucleotide variants (SNVs) phylogenetic analysis revealed that our ST203 isolates clustered with the previously identified ST203 Lebanese genomes. One exception was isolate EF142, which clustered separately. Similarly, the ST612 isolates displayed close genetic relatedness, highlighting the potential clonal spread and persistence of VRE across different regions in Lebanon (Fig. 1). No data currently exist on the prevalence of CC17 in non-clinical settings in Lebanon because of a lack of studies in this area. However, its global dissemination at the human–animal–environment interface has been well-documented (17–19). This highlights CC17’s relevance in clinical and environmental contexts. It also points to the need for further research using a multifaceted One Health approach to understand its transmission dynamics across sectors in Lebanon. Additionally, identifying a new subtype within the local VRE population confirms the importance of comprehensive molecular surveillance to detect emerging variants with enhanced resistance.

Interestingly, all the isolates harbored several virulence factors (14 to 19) and were classified as potential human pathogens (80%–90%) by PathogenFinder, indicating a strong pathogenic potential, especially for immunocompromised patients at high risk of severe VRE infections (20). The identified genes (Table 1) are mainly involved in adhesion to host tissues, immune evasion, and biofilm formation (21). Notably, the *hyl_Efm_* gene, associated with hyaluronidase activity and often linked to enhanced virulence in *E. faecium* (14), was detected exclusively in the two ST612 genomes.

This study provided critical insights into the genomic features of VRE, emphasizing the alarming clonal dissemination of MDR CC17 strains in Lebanese hospitals and outpatient settings. Although there is concordance between WGS and AST results, phenotypic susceptibility testing remains essential for confirming genomic data and evaluating novel antimicrobials against VRE. Effectively addressing the rising VRE challenge requires integrated One Health strategies, improved healthcare infrastructure, strengthened diagnostic capabilities, and targeted infection control measures to promote health outcomes, particularly in disenfranchised communities in low-income countries that can serve as potential hotspots for the emergence of AMR strains. The emergence and spread of AMR in Lebanon, a country facing numerous challenges, serves as a reminder of the heightened risk of AMR in low-income countries and the need to invest in these countries to control the spread of AMR locally and globally.

## Data Availability

Short-read sequence data were submitted to the European Nucleotide Archive (ENA, https://www.ebi.ac.uk/ena/) under study number PRJNA1039514. All accession numbers of the genomes used in this study are listed in Table S3.

## SUPPLEMENTAL MATERIAL

Supplemental material spectrum.00171-25-s0001.docx Additional data.

Supplemental tables spectrum.00171-25-s0002.docx Tables S1 to S3.

ASM does not own the copyrights to Supplemental Material that may be linked to, or accessed through, an article. The authors have granted ASM a non-exclusive, world-wide license to publish the Supplemental Material files. Please contact the corresponding author directly for reuse.
