# Supplementary material for: Critical genomic insights into vancomycin-resistant Enterococcus faecium in Lebanon
Source: Microbiol Spectr. 2025 Aug 5;13(9):e00171-25. doi: 10.1128/spectrum.00171-25 (PMC12403557; doi:10.1128/spectrum.00171-25)
Supplement: Supplemental material — Additional data. [file spectrum.00171-25-s0001.docx]

**Supplementary text**

**Genome Sequencing and processing**

Total DNA was extracted with the QiaAmp DNA Mini kit (Qiagen, Germantown, USA) from overnight cultures in Brain Heart Infusion Agar (Difco, USA) at 37 °C. Whole genome sequencing (WGS) was performed using the Illumina NGS platforms and various bioinformatics tools were applied to investigate the isolates' resistome, virulome, pathogenicity, and multi-locus sequence types (MLST). Pair-ended short-read Illumina^®^ Miseq sequencing was performed as described previously (1). After sequencing, raw reads were subjected to quality control using fastp (v0.23.2) to remove low-quality bases and reads (2). The following parameters were applied: --qualified_quality_phred 30, --unqualified_percent_limit 10, and --length_required 50. High-quality reads were then used for de novo genome assembly using shovill (v1.1.0) with default parameters (https://github.com/tseemann/shovill).

**Bioinformatic Analyses**

The Multi-Locus Sequence Typing (MLST) profiles were determined using mlst (v2.19.0) (https://github.com/tseemann/mlst). One genome could not be assigned a sequence type (ST) due to the presence of a novel allele in the *gyd* gene; as a result, it was submitted to PubMLST for curation and assignment (3) **(Supplementary Table 1)**. Antimicrobial resistance genes were identified through ResFinder (v4.5.0) using default cutoffs (90% minimum identity and 60% minimum coverage) (4). Virulence factors were identified through VirulenceFinder (V2.0) using 100% minimum identity and 100% minimum coverage (5, 6). Plasmid types were detected using PlasmidFinder (v2.1) with the default thresholds of 95% minimum identity and 60% minimum coverage (7).

Parsnp (v1.7.4) (http://harvest.readthedocs.io/en/latest/content/parsnp.html) was used to align the core genomes of *E. faecium* strains from the present study (n = 9) against the reference genome (acc no. GCA_003020745.1) (8, 9). Whole genome sequences of *E. faecium* strains previously isolated in Lebanon and available in PubMLST were also included in the analysis (3). A maximum-likelihood phylogenetic tree was then generated using RAxML (v8.2.12), embedded within Parsnp (10). The final phylogenetic tree was visualized and annotated using ggtree (v3.10.1) and was mid-point rooted (11).

**References**

1. Kassem, II, Wang J, Gorbani Tajani A, Esseili MA, Hassan J, Yassine I, Osman M, Bisha B. 2024. Draft genome sequences of antibiotic-resistant *Serratia* and *Enterobacter* species isolated from imported fresh produce in Georgia, USA. Microbiol Resour Announc 13:e0113923.

2. Chen S, Zhou Y, Chen Y, Gu J. 2018. fastp: an ultra-fast all-in-one FASTQ preprocessor. Bioinformatics 34:i884-i890.

3. Jolley KA, Bray JE, Maiden MCJ. 2018. Open-access bacterial population genomics: BIGSdb software, the PubMLST.org website and their applications. Wellcome Open Res 3:124.

4. Bortolaia V, Kaas RS, Ruppe E, Roberts MC, Schwarz S, Cattoir V, Philippon A, Allesoe RL, Rebelo AR, Florensa AF, Fagelhauer L, Chakraborty T, Neumann B, Werner G, Bender JK, Stingl K, Nguyen M, Coppens J, Xavier BB, Malhotra-Kumar S, Westh H, Pinholt M, Anjum MF, Duggett NA, Kempf I, Nykäsenoja S, Olkkola S, Wieczorek K, Amaro A, Clemente L, Mossong J, Losch S, Ragimbeau C, Lund O, Aarestrup FM. 2020. ResFinder 4.0 for predictions of phenotypes from genotypes. J Antimicrob Chemother 75:3491-3500.

5. Joensen KG, Scheutz F, Lund O, Hasman H, Kaas RS, Nielsen EM, Aarestrup FM. 2014. Real-time whole-genome sequencing for routine typing, surveillance, and outbreak detection of verotoxigenic *Escherichia coli*. J Clin Microbiol 52:1501-10.

6. Malberg Tetzschner AM, Johnson JR, Johnston BD, Lund O, Scheutz F. 2020. In silico genotyping of *Escherichia coli* isolates for extraintestinal virulence genes by use of whole-genome sequencing data. J Clin Microbiol 58:e01269-20.

7. Carattoli A, Hasman H. 2020. PlasmidFinder and in silico pMLST: Identification and typing of plasmid replicons in whole-genome sequencing (WGS). Methods Mol Biol 2075:285-294.

8. Treangen TJ, Ondov BD, Koren S, Phillippy AM. 2014. The Harvest suite for rapid core-genome alignment and visualization of thousands of intraspecific microbial genomes. Genome Biol 15:524.

9. Lee RS, Goncalves da Silva A, Baines SL, Strachan J, Ballard S, Carter GP, Kwong JC, Schultz MB, Bulach DM, Seemann T, Stinear TP, Howden BP. 2018. The changing landscape of vancomycin-resistant *Enterococcus faecium* in Australia: a population-level genomic study. J Antimicrob Chemother 73:3268-3278.

10. Stamatakis A. 2014. RAxML version 8: a tool for phylogenetic analysis and post-analysis of large phylogenies. Bioinformatics 30:1312-3.

11. Yu G, Smith DK, Zhu H, Guan Y, Lam TT-Y. 2017. ggtree: an r package for visualization and annotation of phylogenetic trees with their covariates and other associated data. Methods Ecol Evol 8:28-36.
