# Supplementary material for: Critical genomic insights into vancomycin-resistant Enterococcus faecium in Lebanon
Source: Microbiol Spectr. 2025 Aug 5;13(9):e00171-25. doi: 10.1128/spectrum.00171-25 (PMC12403557; doi:10.1128/spectrum.00171-25)
Supplement: Supplemental tables — Tables S1 to S3. [file spectrum.00171-25-s0002.docx]

**Supplementary Table 1.** Characteristics of hospitalized patients with vancomycin-resistant *Enterococcus faecium* (VRE).

| **Patient ID** | **Age (years)** | **Sex** | **Origin of VRE** | **Previous antimicrobial treatment** | **Nosocomial acquisition** | **Date of isolation (DD/MM/YY)** | **COVID-19** | **Department** | **Outcome** |
| --- | --- | --- | --- | --- | --- | --- | --- | --- | --- |
| EF142 | 63 | M | Rectal | No | No | 03/11/19 | - | Cardiology | Recovery |
| EF321 | 78 | F | Rectal | No | No | 23/09/20 | + | Cardiology | Recovery |
| EF901 | 84 | F | Rectal | No | No | 01/09/21 | - | Cardiology | Death |
| EF1180 | 75 | M | Rectal | Yes | Yes (30 days after admission) | 09/02/22 | + | Intensive care unit | Death |
| EF1196 | 77 | M | Rectal | No | No | 02/03/22 | - | Urology | Recovery |
| EF1261 | 98 | F | Rectal | No | No | 19/04/22 | + | Intensive care unit | Recovery |
| EF1276 | 96 | F | Rectal | No | No | 07/04/22 | - | Intensive care unit | Recovery |
| EF1334 | 86 | F | Urine | No | No | 07/06/22 | - | Cardiology | Recovery |
| EF1337 | 77 | F | Axillary | No | No | 11/06/22 | - | Cardiology | Recovery |

**Supplementary Table 2.** Antimicrobial resistance patterns of vancomycin-resistant *Enterococcus faecium* clinical isolates recovered from hospitalized patients in Lebanon. The antimicrobials are arranged according to the order of antimicrobials/classes listed in the CLSI guidelines.

| **Antimicrobial agent** | **Antimicrobial susceptibility method** | **EF 142** | **EF 321** | **EF 901** | **EF 1180** | **EF 1196** | **EF 1261** | **EF 1276** | **EF 1334** | **EF 1337** |
| --- | --- | --- | --- | --- | --- | --- | --- | --- | --- | --- |
| Ampicillin | Disc diffusion | 6 (R) | 6 (R) | 6 (R) | 6 (R) | 6 (R) | 6 (R) | 6 (R) | 6 (R) | 6 (R) |
| Vancomycin | Disc diffusion | 6 (R) | 6 (R) | 6 (R) | 6 (R) | 6 (R) | 6 (R) | 6 (R) | 6 (R) | 6 (R) |
| Dalbavancin | E-test | > 32 (R) | > 32 (R) | 0.19 (S) | > 32 (R) | > 32 (R) | > 32 (R) | > 32 (R) | 1.5 (R) | > 32 (R) |
| Telavancin | E-test | 2 (R) | 1.5 (R) | 0.38 (R) | 1.5 (R) | 4 (R) | 4 (R) | 2 (R) | 0.38 (R) | 3 (R) |
| Teicoplanin | E-test | > 256 (R) | 64 (R) | 8 (S) | 48 (R) | > 32 (R) | > 256 (R) | 32 (R) | 24 (IR) | > 256 (R) |
| Daptomycin | E-test | 1 (SDD) | 1.5 (SDD) | 1 (SDD) | 0.75 (SDD) | 2 (SDD) | 1.5 (SDD) | 1.5 (SDD) | 1.5 (SDD) | 1.5 (SDD) |
| Erythromycin | Disc diffusion | 6 (R) | 6 (R) | 6 (R) | 6 (R) | 6 (R) | 6 (R) | 6 (R) | 6 (R) | 6 (R) |
| Tetracycline | Disc diffusion | 6 (R) | 6 (R) | 6 (R) | 6 (R) | 6 (R) | 25 (S) | 6 (R) | 6 (R) | 6 (R) |
| Doxycycline | Disc diffusion | 6 (R) | 6 (R) | 6 (R) | 12 (R) | 10 (R) | 25 (S) | 10 (R) | 6 (R) | 6 (R) |
| Tigecycline^§^ | Disc diffusion | 21 (R) | 20 (R) | 17 (R) | 20 (R) | 23 (S) | 24 (S) | 23 (S) | 20 (R) | 17 (R) |
| Eravacycline^§^ | Disc diffusion | 20 (R) | 18 (R) | 18 (R) | 22 (R) | 24 (S) | 24 (S) | 24 (S) | 22 (R) | 21 (R) |
| Levofloxacin | Disc diffusion | 6 (R) | 6 (R) | 6 (R) | 6 (R) | 6 (R) | 6 (R) | 6 (R) | 6 (R) | 6 (R) |
| Norfloxacin | Disc diffusion | 6 (R) | 6 (R) | 6 (R) | 6 (R) | 6 (R) | 6 (R) | 6 (R) | 6 (R) | 6 (R) |
| Nitrofurantoin | Disc diffusion | 6 (R) | 6 (R) | 6 (R) | 6 (R) | 6 (R) | 6 (R) | 6 (R) | 6 (R) | 6 (R) |
| Fosfomycin | Disc diffusion | 17 (S) | 16 (S) | 12 (R) | 15 (IR) | 15 (IR) | 16 (S) | 15 (IR) | 13 (IR) | 16 (S) |
| Chloramphenicol | Disc diffusion | 13 (IR) | 15 (IR) | 24 (S) | 22 (S) | 23 (S) | 16 (IR) | 25 (S) | 21 (S) | 15 (IR) |
| Quinupristin-dalfopristin | E-test | 1.5 (IR) | 1 (S) | 0.25 (S) | 1 (S) | 1.5 (IR) | 1 (S) | 0.5 (S) | 0.5 (S) | 0.5 (S) |
| Linezolid | Disc diffusion | 23 (S) | 25 (S) | 24 (S) | 24 (S) | 24 (S) | 23 (S) | 24 (S) | 23 (S) | 25 (S) |
| Gentamicin | E-test | > 500 (R) | > 500 (R) | > 500 (R) | > 500 (R) | > 500 (R) | > 500 (R) | > 500 (R) | > 500 (R) | < 500 (S) |

^1^Resistance to antimicrobials was determined using the disc diffusion and E-test assays according to the Clinical and Laboratory Standards Institute (CLSI-M100) guidelines (<https://clsi.org/standards/products/microbiology/companion/using-m100/>). R, resistance (red cells); IR, intermediate resistance (orange cells); SDD, susceptible-dose dependent (white cells); S, susceptible (white cells).

^§^The CLSI guidelines do not provide clinical breakpoints for tigecycline and eravacycline; therefore, the clinical breakpoints were adopted from the European Committee on Antimicrobial Susceptibility Testing (EUCAST) guidelines.

**Supplementary Table 3.** Accession numbers of genomes used in this study

| **Genome ID** | **BioSample accession number** | **Reference** |
| --- | --- | --- |
| EF1180 | [SAMN38220490](https://www.ncbi.nlm.nih.gov/biosample/SAMN38220490/) | This study |
| EF1196 | [SAMN38220491](https://www.ncbi.nlm.nih.gov/biosample/SAMN38220491/) | This study |
| EF1261 | [SAMN38220492](https://www.ncbi.nlm.nih.gov/biosample/SAMN38220492/) | This study |
| EF1276 | [SAMN38220493](https://www.ncbi.nlm.nih.gov/biosample/SAMN38220493/) | This study |
| EF1334 | [SAMN38220494](https://www.ncbi.nlm.nih.gov/biosample/SAMN38220494/) | This study |
| EF1337 | [SAMN38220495](https://www.ncbi.nlm.nih.gov/biosample/SAMN38220495/) | This study |
| EF142 | [SAMN38220496](https://www.ncbi.nlm.nih.gov/biosample/SAMN38220496/) | This study |
| EF321 | [SAMN38220497](https://www.ncbi.nlm.nih.gov/biosample/SAMN38220497/) | This study |
| EF901 | [SAMN38220498](https://www.ncbi.nlm.nih.gov/biosample/SAMN38220498/) | This study |
| AUSMDU00004024 | [SAMN08628409](https://www.ncbi.nlm.nih.gov/biosample/?term=SAMN08628409) | (1) |
| VRE_6 | [SAMN14073378](https://www.ncbi.nlm.nih.gov/biosample/SAMN14073378) | PubMLST database |
| VRE_8 | [SAMN14073380](https://www.ncbi.nlm.nih.gov/biosample/SAMN14073380) | PubMLST database |
| VRE_1 | [SAMN14073373](https://www.ncbi.nlm.nih.gov/biosample/SAMN14073373) | PubMLST database |
| VRE_10 | [SAMN14073382](https://www.ncbi.nlm.nih.gov/biosample/SAMN14073382) | PubMLST database |
| VRE_11 | [SAMN14073383](https://www.ncbi.nlm.nih.gov/biosample/SAMN14073383) | PubMLST database |
| VRE_12 | [SAMN14073384](https://www.ncbi.nlm.nih.gov/biosample/SAMN14073384) | PubMLST database |
| VRE_13 | [SAMN14073385](https://www.ncbi.nlm.nih.gov/biosample/SAMN14073385) | PubMLST database |
| VRE_14 | [SAMN14073386](https://www.ncbi.nlm.nih.gov/biosample/SAMN14073386) | PubMLST database |
| VRE_15 | [SAMN14073387](https://www.ncbi.nlm.nih.gov/biosample/SAMN14073387) | PubMLST database |
| VRE_16 | [SAMN14073388](https://www.ncbi.nlm.nih.gov/biosample/SAMN14073388) | PubMLST database |
| VRE_17 | [SAMN14073389](https://www.ncbi.nlm.nih.gov/biosample/SAMN14073389) | PubMLST database |
| VRE_18 | [SAMN14073390](https://www.ncbi.nlm.nih.gov/biosample/SAMN14073390) | PubMLST database |
| VRE_19 | [SAMN14073391](https://www.ncbi.nlm.nih.gov/biosample/SAMN14073391) | PubMLST database |
| VRE_2 | [SAMN14073374](https://www.ncbi.nlm.nih.gov/biosample/SAMN14073374) | PubMLST database |
| VRE_20 | [SAMN14073392](https://www.ncbi.nlm.nih.gov/biosample/SAMN14073392) | PubMLST database |
| VRE_21 | [SAMN14073393](https://www.ncbi.nlm.nih.gov/biosample/SAMN14073393) | PubMLST database |
| VRE_22 | [SAMN14073394](https://www.ncbi.nlm.nih.gov/biosample/SAMN14073394) | PubMLST database |
| VRE_23 | [SAMN14073395](https://www.ncbi.nlm.nih.gov/biosample/SAMN14073395) | PubMLST database |
| VRE_24 | [SAMN14073396](https://www.ncbi.nlm.nih.gov/biosample/SAMN14073396) | PubMLST database |
| VRE_25 | [SAMN14073397](https://www.ncbi.nlm.nih.gov/biosample/SAMN14073397) | PubMLST database |
| VRE_26 | [SAMN14073398](https://www.ncbi.nlm.nih.gov/biosample/SAMN14073398) | PubMLST database |
| VRE_27 | [SAMN14073399](https://www.ncbi.nlm.nih.gov/biosample/SAMN14073399) | PubMLST database |
| VRE_28 | [SAMN14073400](https://www.ncbi.nlm.nih.gov/biosample/SAMN14073400) | PubMLST database |
| VRE_29 | [SAMN14073401](https://www.ncbi.nlm.nih.gov/biosample/SAMN14073401) | PubMLST database |
| VRE_3 | [SAMN14073375](https://www.ncbi.nlm.nih.gov/biosample/SAMN14073375) | PubMLST database |
| VRE_30 | [SAMN14073402](https://www.ncbi.nlm.nih.gov/biosample/SAMN14073402) | PubMLST database |
| VRE_31 | [SAMN14073403](https://www.ncbi.nlm.nih.gov/biosample/SAMN14073403) | PubMLST database |
| VRE_32 | [SAMN14073404](https://www.ncbi.nlm.nih.gov/biosample/SAMN14073404) | PubMLST database |
| VRE_33 | [SAMN14073405](https://www.ncbi.nlm.nih.gov/biosample/SAMN14073405) | PubMLST database |
| VRE_35 | [SAMN14073407](https://www.ncbi.nlm.nih.gov/biosample/SAMN14073407) | PubMLST database |
| VRE_36 | [SAMN14073408](https://www.ncbi.nlm.nih.gov/biosample/SAMN14073408) | PubMLST database |
| VRE_37 | [SAMN14073409](https://www.ncbi.nlm.nih.gov/biosample/SAMN14073409) | PubMLST database |
| VRE_38 | [SAMN14073410](https://www.ncbi.nlm.nih.gov/biosample/SAMN14073410) | PubMLST database |
| VRE_39 | [SAMN14073411](https://www.ncbi.nlm.nih.gov/biosample/SAMN14073411) | PubMLST database |
| VRE_4 | [SAMN14073376](https://www.ncbi.nlm.nih.gov/biosample/SAMN14073376) | PubMLST database |
| VRE_40 | [SAMN14073412](https://www.ncbi.nlm.nih.gov/biosample/SAMN14073412) | PubMLST database |
| VRE_41 | [SAMN14073413](https://www.ncbi.nlm.nih.gov/biosample/SAMN14073413) | PubMLST database |
| VRE_42 | [SAMN14073414](https://www.ncbi.nlm.nih.gov/biosample/SAMN14073414) | PubMLST database |
| VRE_43 | [SAMN14073415](https://www.ncbi.nlm.nih.gov/biosample/SAMN14073415) | PubMLST database |
| VRE_44 | [SAMN14073416](https://www.ncbi.nlm.nih.gov/biosample/SAMN14073416) | PubMLST database |
| VRE_45 | [SAMN14073417](https://www.ncbi.nlm.nih.gov/biosample/SAMN14073417) | PubMLST database |
| VRE_46 | [SAMN14073418](https://www.ncbi.nlm.nih.gov/biosample/SAMN14073418) | PubMLST database |
| VRE_47 | [SAMN14073419](https://www.ncbi.nlm.nih.gov/biosample/SAMN14073419) | PubMLST database |
| VRE_48 | [SAMN14073420](https://www.ncbi.nlm.nih.gov/biosample/SAMN14073420) | PubMLST database |
| VRE_5 | [SAMN14073377](https://www.ncbi.nlm.nih.gov/biosample/SAMN14073377) | PubMLST database |
| VRE_50 | [SAMN14073422](https://www.ncbi.nlm.nih.gov/biosample/SAMN14073422) | PubMLST database |
| VRE_51 | [SAMN14073423](https://www.ncbi.nlm.nih.gov/biosample/SAMN14073423) | PubMLST database |
| VRE_52 | [SAMN14073424](https://www.ncbi.nlm.nih.gov/biosample/SAMN14073424) | PubMLST database |
| VRE_53 | [SAMN14073425](https://www.ncbi.nlm.nih.gov/biosample/SAMN14073425) | PubMLST database |
| VRE_54 | [SAMN14073426](https://www.ncbi.nlm.nih.gov/biosample/SAMN14073426) | PubMLST database |
| VRE_56 | [SAMN14073428](https://www.ncbi.nlm.nih.gov/biosample/SAMN14073428) | PubMLST database |
| VRE_7 | [SAMN14073379](https://www.ncbi.nlm.nih.gov/biosample/SAMN14073379) | PubMLST database |
| VRE_9 | [SAMN14073381](https://www.ncbi.nlm.nih.gov/biosample/SAMN14073381) | PubMLST database |

**References**

1. Lee RS, Goncalves da Silva A, Baines SL, Strachan J, Ballard S, Carter GP, Kwong JC, Schultz MB, Bulach DM, Seemann T, Stinear TP, Howden BP. 2018. The changing landscape of vancomycin-resistant *Enterococcus faecium* in Australia: a population-level genomic study. J Antimicrob Chemother 73:3268-3278.
